# The economic burden of human papillomavirus-related precancers and cancers in Sweden

**DOI:** 10.1371/journal.pone.0179520

**Published:** 2017-06-26

**Authors:** Ellinor Östensson, Maria Silfverschiöld, Lennart Greiff, Christine Asciutto, Johan Wennerberg, Marie-Louise Lydryp, Ulf Håkansson, Pär Sparén, Christer Borgfeldt

**Affiliations:** 1 Department of Medical Epidemiology and Biostatistics, Karolinska Institutet, Stockholm, Sweden; 2 Department of Women´s and Children´s Health, Division of Obstetrics and Gynecology, Karolinska Institutet, Stockholm, Sweden; 3 Department of Otorhinolaryngology Head & Neck Surgery, Skånes University Hospital, Lund University, Lund, Sweden; 4 Department of Obstetrics and Gynecology, Skånes University Hospital, Lund University, Lund, Sweden; 5 Department of Experimental Medical Science, Lund University, Lund, Sweden; 6 Department of Urology, Skånes University Hospital, Lund University, Malmö, Sweden; Brigham and Women's Hospital, UNITED STATES

## Abstract

**Background:**

High-risk (HR) human papillomavirus (HPV) infection is an established cause of malignant disease. We used a societal perspective to estimate the cost of HR HPV-related cervical, vulvar, vaginal, anal, and penile precancer and cancer, and oropharyngeal cancer in Sweden in 2006, 1 year before HPV vaccination became available in the country.

**Materials and methods:**

This prevalence-based cost-of-illness study used diagnosis-specific data from national registries to determine the number of HR HPV-related precancers and cancers. The HR HPV-attributable fractions of these diseases were derived from a literature review and applied to the total burden to estimate HR HPV-attributable costs. Direct costs were based on health care utilization and indirect costs on loss of productivity due to morbidity (i.e., sick leave and early retirement) and premature mortality.

**Results:**

The total annual cost of all HR HPV-attributable precancers and cancers was €94 million (€10.3/inhabitant). Direct costs accounted for €31.3 million (€3.4/inhabitant) of the total annual cost, and inpatient care amounted to €20.7 million of direct costs. Indirect costs made up €62.6 million (€6.9/inhabitant) of the total annual cost, and premature mortality amounted to €36 million of indirect costs. Cervical precancer and cancer was most costly (total annual cost €58.4 million). Among cancers affecting both genders, anal precancer and cancer, and oropharyngeal cancer were the most costly (€11.2 million and €11.9 million, respectively). For oropharyngeal cancer, males had the highest health care utilization and represented 71% of the total annual cost. Penile precancer and cancer was least costly (€2.6 million).

**Conclusion:**

The economic burden of HR HPV-related precancers and cancers is substantial. The disease-related management and treatment costs we report are relevant as a point of reference for future economic evaluations investigating the overall benefits of HPV vaccination in females and males in Sweden.

## Introduction

Infection with high-risk (HR) human papillomavirus (HPV) types is the established cause of several significant diseases, namely precancer and cancer of the cervix, vulva, vagina, anus, penis, and head and neck (oral and oropharynx) [1]. Worldwide, HR HPV16 and 18 account for about 70% of all cases of cervical cancer and cervical dysplasia [2, 3]. For head and neck cancer, HR HPV types (particularly HPV16) have been linked to oral and oropharyngeal cancer, and the highest HR HPV prevalence has been found in the tonsils, with HPV DNA being present in around 45–70% of cases [4]. Low-risk HPV6 and 11 cause benign genital warts [5] and recurrent respiratory papillomatosis [6]. HPV DNA is detected in around half of penile cancers, with HPV16 being the most common type, followed by HPV18 [7]. Incidence rates of HPV-positive oropharyngeal cancer are increasing and are significantly higher among males than females [8, 9]. The increasing trend in the incidence of and mortality from oropharyngeal cancer among males is similar in most Western countries [10, 11].

Two prophylactic HPV vaccines became available in Sweden in a 2-year period: one bivalent vaccine in 2007 and one quadrivalent (HPV-4) vaccine in 2008. In 2010, the public health policy decision was made to use the HPV-4 vaccine in the HPV Immunization Programme in Sweden. Within the framework of this program, the HPV-4 vaccine was administered in schools to all girls aged 10–12 years [12]. This policy decision was guided by a previous cost-effectiveness analysis carried out by The Swedish Dental and Pharmaceutical Benefits Agency, which confirmed that HPV vaccination of girls in this age group was cost-effective in the prevention of cervical cancer. However, there are no reports on the cost-effectiveness of HPV vaccination in the prevention of non-cervical HPV-related diseases, many of which affect both genders.

HPV-4 vaccination has already been proven effective against HPV infection at the vulva and vagina in females, and at the external genital skin and anal canal in females and males [13–15]. Now the question has become: how should the resources available be used? Is adding males to the HPV Immunization Programme (i.e., creating a gender-neutral vaccination program) more advantageous than other interventions that are currently under review by the Swedish National Board of Health and Welfare to lower HPV infections and related diseases? Economic evaluations look at both the costs and consequences of these issues, and it is this combination that allows the relevant authorities to reach policy decisions [16]. An important step in comparative analyses is to identify relevant costs that arise from resource utilization in the health care sector and other sectors, informal care, and changes in productivity due to work absence. Cost-of-illness studies identify relevant costs and where they occur in a society, and can act as point of reference for further economic evaluations [17]. For diseases such as cancer, cost-of-illness studies can show the distribution of costs to the health care system, social services, and patients themselves, as well as costs related to productivity losses that occur due to the disease or its treatment. This information in turn helps inform policy decision-makers gain knowledge on where the majority of the resources in the health care sector and other sectors are utilized.

According to a recent Swedish cost-of-illness study, the total societal cost for cervical cancer and genital warts in 2009 was €107 million, of which almost 80% were health care utilization costs [18]. However, at present there is no published cost-of-illness study from Sweden estimating the societal costs associated with non-cervical HPV-related diseases.

In order to make future decisions on public health policy, health care budgets, and the eventual modification of the HPV Immunization Programme to a gender-neutral vaccination program, it is important to identify and estimate the relevant costs of all HR HPV-related diseases for both genders. Therefore, the objective of this study was to estimate the costs associated with HR HPV-related cervical, vulvar, vaginal, anal, and penile precancers and cancers, and tonsillar and base of tongue cancer (i.e., oropharyngeal cancers) in Sweden in 2006, 1 year before HPV vaccines became available in the country.

## Materials and methods

### Method of costing

We performed a prevalence-based cost-of-illness study, taking into account all diagnosis-specific events during a given year and all resources utilized or lost as a result of morbidity and mortality [17]. We used national registries to determine the number of patients and events in the health care sector and other sectors to estimate direct costs associated with inpatient and outpatient health care events (somatic health care events/admissions performed in a hospital setting, including admissions for bed days, i.e., inpatient care; and daily health care services administered in a hospital, i.e., outpatient care). National registries and statistical databases were also used to estimate indirect costs (loss of productivity due to morbidity, i.e., sick leave and early retirement, and premature mortality). There is no national registry in Sweden that covers daily health care services for basic medical needs delivered in a primary care setting or by general practitioners without specialized health care resources found at hospitals; therefore this type of care was excluded from this study.

The study population was identified in national registries based on the following codes from the International Classification of Diseases, Revision 10: dysplasia, carcinoma *in situ* (CIS), and cancer of the cervix (N87, D06, C53), vulva (N90, D07.1, C51), vagina (N89, D07.2, C52), and anus (K62, D01.3, C21); CIS and cancer of the penis (D07.4, C60), and cancer of the tonsil (C09) and base of tongue (C01) (i.e., oropharyngeal cancers) (see S1 Table for description of diagnoses). The total number of inpatient and outpatient health care events in 2006 due to these specific diagnoses was extracted from the Swedish National Board of Health and Welfare Inpatient Registry, which contains information on main diagnoses, gender, age, and health care event codes, with an under-reporting rate of less than 1%. Other national registries used in the study were the Swedish Cancer Registry and the Swedish National Quality Registry for Cervical Cancer Prevention, from which information on diagnosis-specific health care events was collected, and the Swedish Social Insurance Agency Registry, from which information on diagnosis-specific sick leave and early retirement days were collected.

### The HR HPV-attributable fraction

To estimate the diagnosis-specific resources utilized or lost as a result of morbidity and mortality attributable to HR HPV, we performed a literature review to identify secondary data available on prevalence rates of HR HPV16, 18, 31, 33, 45, 52, and 58. We performed a systematic literature search in the electronic databases MEDLINE (PubMed), EMBASE (on the Elsevier platform www.embase.com), The World Health Organization, the Institut Català d'Oncologia Information Centre on HPV and Cancer [19], and the International Agency for Research on Cancer [20]. The search years were set to 2000–2015. Two researchers screened the resultant articles, including titles and abstracts of all identified data sources. To be considered, studies had to 1) have epidemiological data derived from population-based databases, registries, or national surveys OR clinical data with HR HPV prevalence reported for individual cancer sites; and 2) report Swedish data relevant to the cancers included in this cost-of-illness study. If these criteria were met, full-text articles were read, discussed, and selected based on consensus among the clinical experts and co-authors in this study (S1 Fig).

Table 1 shows the prevalence rates of the HR HPV types reported in the articles from the literature review. Due to the number of diseases and the heterogeneity of these prevalence rates, we did not standardize prevalence rates or perform any meta-analyses.

**Table 1:** HR HPV* -attributable fraction reported in the articles from the literature review by precancer and cancer site.

Clinical conditions	HR HPV fraction used to calibrate base-case attributable costs	Lower bound(95% CI)	Upper bound(95% CI)	Reference	Range of HR HPV fraction identified in the literature review		References
					Lowest	Highest	
Cervical dysplasia and CIS	72%	-	-	[21]	72%	80%	[21, 22]
Cervical cancer	93%	-	-	[23]	71%	95%	[23–28]
Vulvar dysplasia and CIS	85%	-	-	[29]	73%	100%	[30–32]
Vulvar cancer	31%	24%	39%	[29]	31%	100%	[31, 33–39]
Vaginal dysplasia and CIS	91%	-	-	[29]	60%	100%	[30, 40, 41]
Vaginal cancer	53%	42%	65%	[29]	52%	89%	[35, 42, 43]
Anal dysplasia and CIS	94%	-	-	[29]	69%	98%	[30, 44–46]
Anal cancer	90%	81%	95%	[29]	83%	100%	[46–48]
Penile dysplasia and CIS	89%	79%	94%	[29]	86%	93%	[49, 50]
Penile cancer	81%	74%	86%	[29]	52%	81%	[50–52]
Oropharyngeal cancer	49%	42%	56%	[29, 53]	40%	89%	[53–58]

*Includes HPV16, 18, 31, 33, 45, 52, and 58.

CIS = carcinoma *in situ*

### Direct costs

Direct costs in this study include resource utilization for inpatient and outpatient health care events associated with the detection, treatment, and follow-up of HR HPV-related precancers and cancers. The Patient-level Clinical Costing method [59], known as cost per patient (CPP) in Sweden, was used to calculate the cost of care for each patient or event. The CPP method is used for the national hospitals and includes variable costs (related to equipment and health care personnel time) and fixed or overhead costs (power, phone, heat, rent, administration, etc.). The CPP method includes costs related to a health care event for a specific patient or a group of patients with a specific diagnosis. Direct costs were based on information from the national database on CPP, estimated as the average cost of each health care event for patients with the relevant specific diagnosis.

### Indirect costs

Swedish guidelines specifically recommend the inclusion of indirect costs in economic analyses [60] using the traditional human capital method, which values lost productivity in terms of gross earnings [61]. We used the human capital method to estimate indirect costs, i.e., costs related to lost productivity due to morbidity (sick leave and early retirement) following a diagnosis of HR HPV-related precancer or cancer and premature mortality following a diagnosis of HR HPV-related cancer. Calculations were performed under the simplifying assumption of full employment until age 65 years, which is the general retirement age in Sweden.

#### Morbidity

To estimate the cost of lost productivity due to sick leave, the total number of workdays lost was multiplied by the average daily income for full-time employees in the public and private sector, plus social security contributions [62]. According to the recommendations, we accounted for equity issues and applied a general gross wage rate for all working individuals, both females and males [16, 63]. The average yearly gross earnings for both genders 2006 was €42 600 (including social security contributions estimated as 31.42% of the total earnings) [62]. Based on 226 workdays for one working year; considering an 8-hour work day, this calibrates to 1808 working hours. Based on these numbers, the cost of a full work day was estimated at €188.

The Swedish Social Insurance Agency maintains a registry of diagnosis-specific data regarding long-term sick leave (episodes of more than 14 days) and early retirement days. However, it does not contain information on shorter sick leave periods, as short-term sick leave is financed by the employer. Therefore, our estimates costs for morbidity are based on 1) long-term sick leave days, 2) early retirement days from the Swedish Social Insurance Agency, and 3) short-term sick leave days calculated as the percentage of sick leave due to the HR HPV-related precancer and cancer in December 2006 multiplied by the total aggregated number of short-term sick leave days for both genders in the same year [62].

#### Mortality

The number of working years lost as a result of premature death due to HR HPV-related cancers was calculated by subtracting age at death from an assumed retirement age of 65 years. We then calculated the average cumulative mortality risk at 0–64 years of age using life tables that present future mortality risk for females and males in the Swedish population [62]. These calculations do not take into account any differences in risk factors between individuals or possible future changes in mortality. The mathematical formula was based on age at premature death divided into thirteen 5-year age groups, the number of premature deaths in every age group, and the average age in each age group (considered to be equal to the midpoint +0.5 years, e.g., 32.5 years for age class 30–34 years). The number of working years lost was then multiplied by the average cumulative mortality risk. The indirect costs of mortality due to HR HPV-related cancers was then calculated by multiplying the number of working years lost due to premature death from these cancers by the discounted annual cost of labor at 3% and 5% [16].

### Estimation of annual costs

To develop an aggregate monetary measure of the burden of HR HPV-related precancers and cancers in 2006, the fraction attributable to HR HPV was applied for each disease. The range of the estimates was calculated using the lowest and highest ranges from the literature review. All identifiable associated costs are included in the estimate and are expressed in as 2006 prices, and were converted from Swedish krona (SEK) to Euro (€) using the average exchange rate for 2006 (€1 = SEK 9).

### Ethics

Approval of the study protocol was obtained by the Ethical Review Board at Karolinska Institute, Stockholm, Sweden (reference numbers 02–556, 98:002, 2011/921-32, 2012/1426-32, 2015/1253-32, 2015/1427-32).

## Results

### Direct costs

#### Outpatient care

There were a total of 41 511 outpatient and inpatient health care events associated with HR HPV-related precancers and cancers registered in the Swedish National Board of Health and Welfare Inpatient Registry and the Swedish Cancer Registry during 2006 for both genders. Females represented 37 302 events, and males represented 4209 events (Table 2). Of the 41 511 events, 37 025 were outpatient health care events, of which 29 594 were estimated to be attributable to HR HPV. Females represented 26 739 of these events, and males represented 2855 events (Table 2).

**Table 2:** Number of registered outpatient and inpatient care health care events, number of HR HPV-attributable events, and average cost per patient presented in 2006 €.

		Females						Males					
ICD-code	Diagnosis	Registeredoutpatient care events	HR HPV-attributable outpatient care events	Averagecost per patient	Registered inpatient care events	HR HPV-attributable inpatient care events	Averagecost per patient	Registered outpatient care events	HR HPV-attributableoutpatient care events	Average cost per patient	Registered inpatient care events	HR HPV-attributable inpatient care events	Average cost per patient
		N	n	€	N	n	€	N	n	€	N	n	€
N87	Cervical dysplasia	13 072	9 412	350	241	174	4 245						
D06	Cervical CIS	4 647	4 322	495	296	275	4 241						
C53	Cervical cancer	4 445	4 134	346	1 612	1 499	6 063						
	Cervix; total	22 164	17 867	384	2 149	1 948	5 643						
N90	Vulvar dysplasia	2 604	2 213	253	82	70	2 550						
D07.1	Vulvar CIS	612	520	408	105	89	3 268						
C51	Vulvar cancer	1 416	439	284	484	150	6 808						
	Vulva; total	4 632	3 173	283	671	309	4 825						
N89	Vaginal dysplasia	3 370	3 067	309	88	80	2 453						
D07.2	Vaginal CIS	110	100	531	15	14	4 532						
C52	Vaginal cancer	348	184	286	133	70	6 689						
	Vagina; total	3 828	3 351	315	236	164	4 444						
K62	Anal dysplasia	856	805	400	89	84	4 843	604	568	381	67	63	6 721
D01.3	Anal CIS	48	45	453	4	4	11 217	19	18	534	4	4	11 217
C21	Anal cancer	1 095	986	468	241	217	10 049	367	330	406	101	91	11 612
	Anus; total	1 999	1 835	438	334	304	8 632	990	916	393	172	158	8 843
D07.4	Penile CIS							160	146	263	9	8	3 717
C60	Penile cancer							784	633	300	174	141	5 993
	Penis; total							944	779	293	183	149	5 871
C09	Tonsillar cancer	632	310	279	155	76	7 781	1 745	855	279	341	167	5 393
C01	Base of tongue cancer	414	203	253	88	43	7 420	621	304	242	157	77	4 944
	Oropharynx; total	1 046	513	269	243	119	7 650	2 366	1 159	269	498	244	5 252
	Total	33 669	26 739	365	3633	2 845	58 89	4 300	2 855	316	853	550	6 678

N = total register-based number, n = prevalence-based number,

CIS = carcinoma *in situ*.

For females, cervical dysplasia represented the majority of HR HPV-attributable outpatient health care events, with 9412 events (i.e., 72% of the 13 072 registered), closely followed by cervical CIS and cervical cancer, with 4322 (i.e., 93% of the 4647 registered) and 4134 (i.e., 93% of the 4445 registered) outpatient health care events, respectively. For vaginal and vulvar dysplasia, there were 3067 (i.e., 91% of the 3370 registered) and 2213 (i.e., 85% of the 2604 registered) HR HPV-attributable outpatient health care events. Altogether, there were 24 391 (i.e., 80% of 30 627 registered) HR HPV-attributable outpatient care health events for all gynecological cancers combined (cervical, vaginal, and vulvar dysplasia, CIS, and cancer). Anal dysplasia, CIS, and cancer accounted for 1835 (i.e., 92% of the 1999 registered) and oropharyngeal cancer accounted for 513 (i.e., 49% of the 1046 registered) HR HPV-attributable outpatient health care events among females.

For males, oropharyngeal cancer represented the majority of HR HPV-attributable outpatient health care events, with 1159 events (i.e., 49% of the 2366 registered). Tonsillar cancer represented 855 (i.e., 49% of the 1745 registered) and base of tongue cancer 304 (i.e., 49% of 621 registered) of these events. HR HPV-attributable outpatient health care events for anal dysplasia, CIS, or cancer and penile CIS and cancer were estimated at 916 (i.e., 93% of the 990 registered) and 776 (i.e., 82% of the 944 registered), respectively.

The average cost for outpatient care events due to HR HPV-attributable cervical dysplasia, CIS, and cancer was €350, €495, and €346, respectively. For both genders with tonsillar cancer, the average cost was €279, while the cost for base of tongue cancer was €253 for females and €242 for males. Average cost for HR HPV-attributable anal dysplasia, CIS, and cancer was €400, €453, and €468, respectively for females and €381, €534, and €406, respectively for males.

#### Inpatient care

There were a total of 4486 inpatient health care events associated with HR HPV-related precancers and cancers registered in both genders, of which 3401 events were estimated to be attributable to HR HPV. Females represented 2845 and males 550 of these HPV-attributable events.

For females, cervical dysplasia, CIS, and cancer made up the majority of HR HPV-attributable inpatient health care events, with a combined total of 1948 (i.e., 91% of the 2149 registered). There were 309 (i.e., 46% of the 671 registered) HR HPV-attributable inpatient health care events for vulvar dysplasia, CIS, and cancer combined, and 304 (i.e., 91% of the 334 registered) events for anal dysplasia, CIS, and cancer combined among females. There were 164 (i.e., 70% of the 236 registered) HR HPV-attributable inpatient health care events for vaginal dysplasia, CIS, and cancer combined, and 119 (i.e., 49% of the 243 registered) for oropharyngeal cancer among females (76 [49% of the 155 registered] for tonsillar cancer and 43 [49% of the 88 registered] for base of tongue cancer).

For males, oropharyngeal cancer had the highest number of HR HPV-attributable inpatient health care events, with a total of 244 (i.e., 49% of the 498 registered), of which there were 167 (i.e., 49% of the 341 registered) and 77 (i.e., 49% of the 157 registered) events for tonsillar and base of tongue cancer, respectively. There were 158 (i.e., 92% of the 172 registered) HR HPV-attributable inpatient health care events for anal dysplasia, CIS, and cancer among males, and 49 (i.e., 81% of the 183 registered) events for penile CIS and cancer.

The average CPP due to HR HPV-attributable inpatient care for cervical cancer was €6063. Average cost for females with tonsillar and base of tongue cancer was €7781 and €7420, respectively, which was 31% and 33% higher than the average cost for males (€5393 and €4944, respectively). The average cost per HR HPV-attributable inpatient health care event due to anal cancer was €10 049 for females and €11 612 for males.

**Fig 1:**
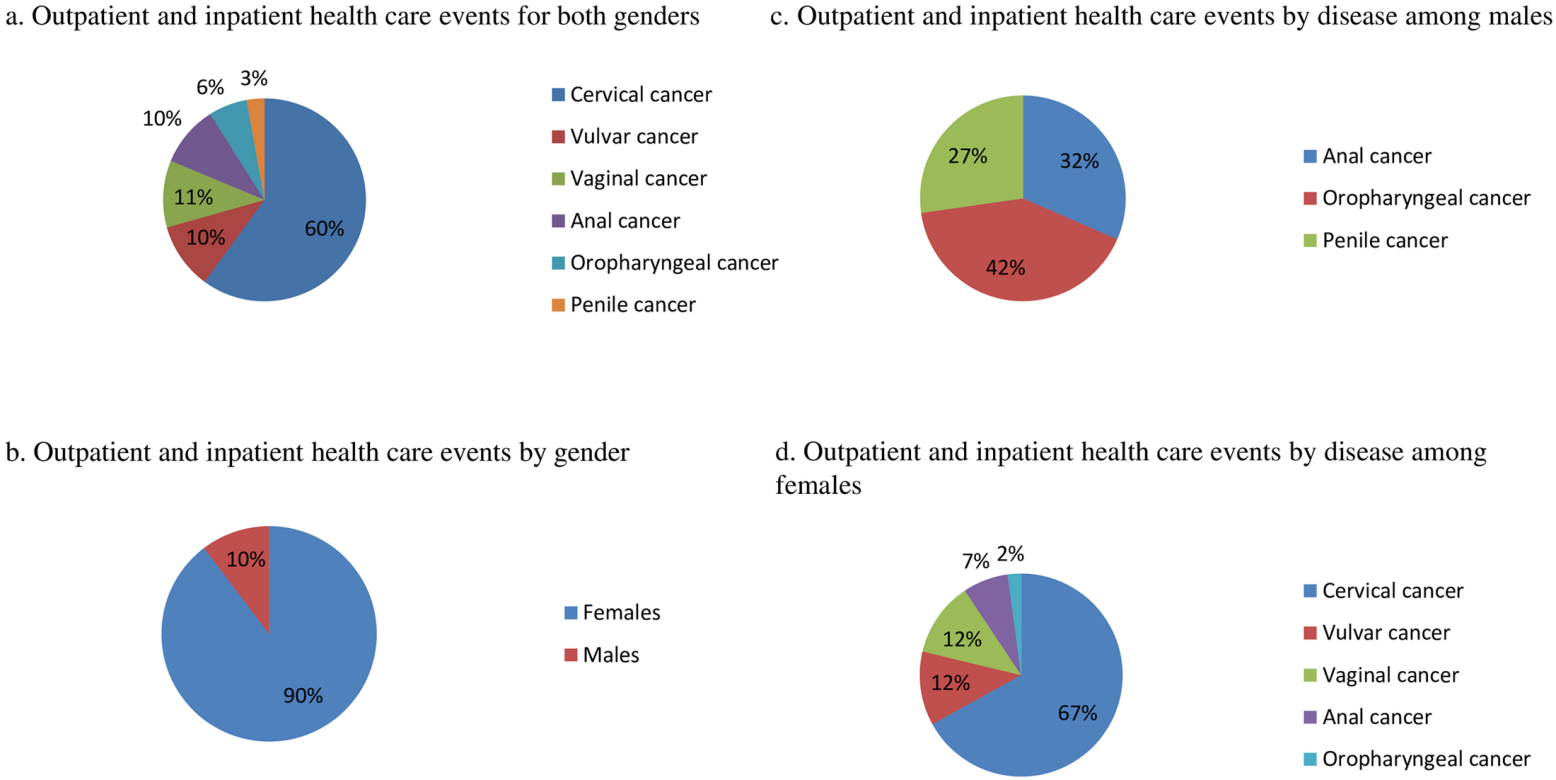
HR HPV-attributable fraction of outpatient and inpatient health care events by high-risk human papillomavirus-related disease and gender. (a) Outpatient and inpatient health care events for both genders. (b). Outpatient and inpatient health care events by gender. (c). Outpatient and inpatient health care events by disease among males (d) Outpatient and inpatient health care events by disease among females.

According to our estimates, cervical cancer was responsible for the largest proportion of all HR HPV-attributable outpatient and inpatient health care events (60%), followed by vaginal (11%), vulvar (10%), anal (10%), oropharyngeal (6%), and penile cancer (3%) (Fig 1a). In total, gynecological cancers represented 81% of all HR HPV-attributable outpatient and inpatient health care events, and females represented 90% of all events (Fig 1b). Among males oropharyngeal cancer was responsible for the largest proportion of HR HPV-attributable outpatient and inpatient health care events (42%), followed by anal (32%) and penile cancer (27%) (Fig 1c). Among females, cervical cancer represented the majority of HR HPV-attributable outpatient and inpatient health care events (67%), followed by vulvar (12%), vaginal (12%), anal (7%), and oropharyngeal cancer (2%) (Fig 1d).

### Indirect costs

#### Morbidity

According to the Swedish Social Insurance Agency Registry, the total number of long-term sick leave days and granted early retirement days in December 2006 was 70 725 880 and 201 912 993, respectively. Of these days, 87 484 (64 051 and 23 433 days for females and males, respectively) were associated with HR HPV-related cancer (Table 3). After applying the prevalence rates from the national summary, 64 484 of these long-term sick days (74%; 51 491 and 12 993 days for females and males, respectively) were estimated to be attributable to HR HPV. The total number of short-term sick leave days for all HR HPV-related cancers in Sweden was 18 439 227 in 2006 for both genders. Assuming that HR HPV-related cancer represents the same share of short-term sick leave as long-term sick leave, we estimated short-term sick leave by taking the same share as for long-term-sick leave and applying the cancer-specific prevalence rate. This led to an estimated 20 199 days (0.11%) of short-term sick leave.

**Table 3:** Number of registered sick leave and early retirement days and HPV-attributable sick leave and early retirement days in 2006, presented in €1000.

Diagnosis	Females						Males						
	TLSL	ALSL	TSSL	ASSL	TERD	AERD	TLSL	ALSL	TSSL	ASSL	TERD	AERD	ATC
	N	n	N	n	N	n	N	n	N	n	N		€
Cervical cancer	41 195	38 311	13 551	12 001	31 770	29 546							15 057
Vulvar cancer	6 712	2 081	736	652	8 249	2 557							997
Vaginal cancer	2 043	1 083	383	339	2 966	1 572							564
Anal cancer	7 458	6 712	2 374	2 103	8 078	7 270	2 688	2 419	856	758	2 465	2 218	4 050
Penile cancer							1 285	1 038	367	325	2 890	2 335	697
Tonsillar cancer	4 534	2 221	786	696	5 835	2 859	16 186	7 931	2 805	2 484	11 663	5 715	4 131
Base of tongue cancer	2 110	1 083	383	339	1 004	492	3 274	1 604	567	503	3 877	1 900	1 104
Total	64 051	51 491	18 213	16 129	57 901	44 296	23 433	12 993	4 596	4 070	20 895	12 168	26 602

N = total register-based number, n = prevalence-based number, TLSL: Total long-term sick leave days, ALSL: Attributable fraction of long-term sick leave days, TSSL: Total short-term sick leave days, ASSL: Attributable fraction of short-term sick leave days, TERD: total early retirement days, AERD: Attributable fraction of early retirement days, ATC: Attributable fraction of cost

During 2006, the total number of early retirement days registered for both genders with the same main diagnosis was 56 464 78 796 (57 901 and 20 895 days for females and males respectively), with 56 464 days (44 296 and 12 168 days for females and males respectively) estimated to be attributable to HR HPV.

**Fig 2:**
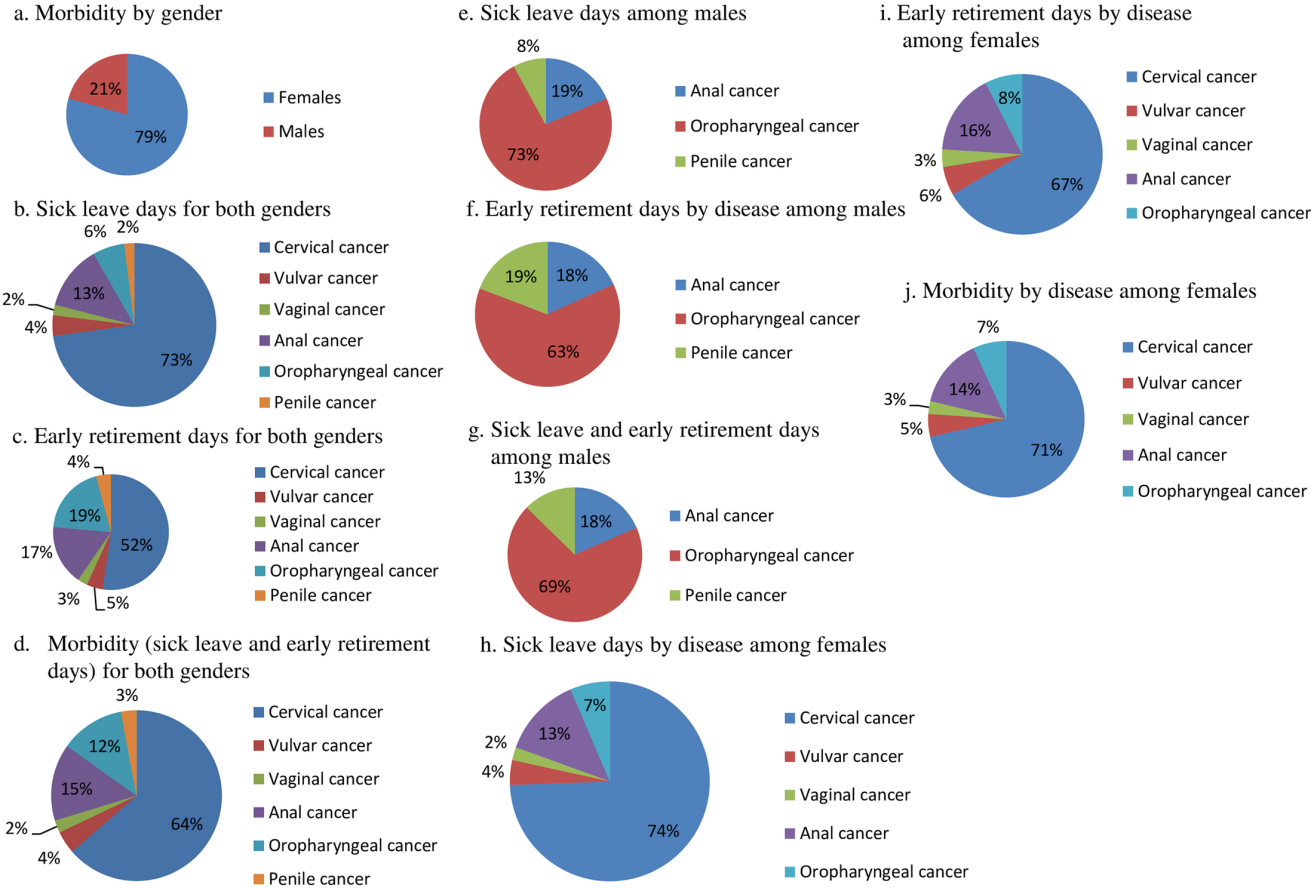
HR HPV-attributable fraction of morbidity (i.e., sick leave and early retirement days) by HPV-related disease and gender. (a) Morbidity by gender. (b) Sick leave days for both genders. (c) Early retirement days for both genders. (d) Morbidity (sick leave and early retirement days) for both genders. (e) Sick leave days among males. (f) Early retirement days by disease among males. (g) Sick leave and early retirement days among males. (h) Sick leave days by disease among females. (i) Early retirement days by disease among females. (j) Morbidity by disease among females.

Females had the largest proportion of HPV-attributable sick leave and early retirement days (i.e., morbidity) in 2006 (79%) (Fig 2a, 2b and 2c). Cervical cancer was responsible for the largest proportion of HPV-attributable morbidity with 64% (Fig 2d). Among males, oropharyngeal cancer represented the majority of HPV-attributable morbidity with 69%, followed by anal cancer with 18%, and penile cancer with 13% (Fig 2e, 2f and 2g). Among females, cervical cancer was responsible for 71% of HPV-attributable morbidity, followed by anal cancer (14%), oropharyngeal cancer (7%), vulvar cancer (5%), and vaginal cancer (3%) (Fig 2h, 2i and 2j).

Three hundred individuals diagnosed with the HPV-related cancers under study were granted early retirement in 2006; the majority was females (73%) between 50–59 years of age. Using the annual labor cost, the total annual cost for morbidity was estimated at €26.6 million.

#### Mortality

According to official statistics from the Swedish National Board of Health and Welfare, 376 individuals died due to HR-HPV related cancers under study in 2006, of which 116 were below the age of 65 years. According to our methodology, only these deaths can be included in our calculations of production loss (Table 4). Cervical cancer was the main cause of death in 56 cases followed by oropharyngeal cancer with 26 deaths, of which 85% were males. For males, oropharyngeal cancer was the main cause of death with 22 deaths, followed by penile and anal cancer, with 6 and 5 deaths, respectively. After applying the HR-HPV prevalence rates, the total number of working years lost due to HR HPV-attributable cancers was estimated at 1071 years (of 1375 totally). Of these years, 944 (88%) were represented by females, and 93% of these were represented by gynecological cancers, followed by anal cancer and oropharyngeal cancer with 4% and 3%, respectively. Of the 127 working years lost due to HR HPV-attributable cancers among males, oropharyngeal cancer represented the majority with 65%, followed by anal and penile cancer with 22% and 13%, respectively. Using a discount rate of 3%, the cost of premature death due to HR HPV-attributable cancers was estimated at €36 million (of €46.8 million totally [3%] and €40.1 million [5%]). The total annual cost decreased by €5.3 million when we applied a 5% discount rate.

**Table 4:** Cost of premature death due to HR HPV-attributable cancers (presented in €1000).

Age groups	Number of registered deaths, F, M (N)	Production loss at 3% discount rate (€)	Production loss at 5% discount rate (€)
20–24	2	1 903	1 409
25–29	1	469	357
30–34	8	5 810	4 554
35–39	9	6 108	4 936
40–44	11	6 335	5 293
45–49	10	3 476	3 013
50–54	14	4 222	3 807
55–59	29	5 793	5 459
60–64	32	1 916	1 880
Total	116	36 031	30 709

N = total register-based number, F = females, M = males.

### Total annual cost

**Fig 3:**
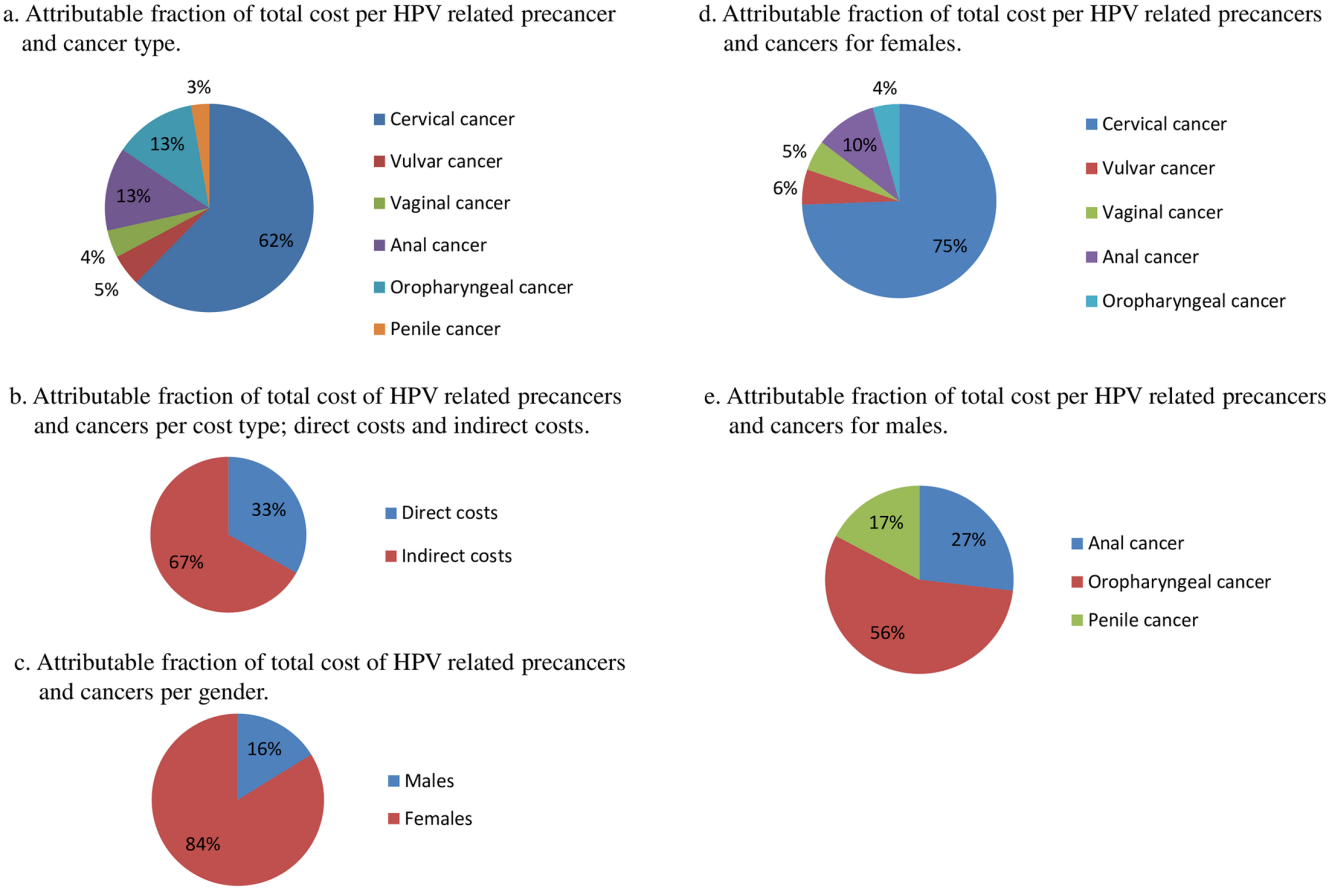
Attributable fraction of total costs including both direct costs (i.e., inpatient care and outpatient care) and indirect costs (i.e., mortality, sick leave, and early retirement days) presented per HPV-related disease and gender. (a) Attributable fraction of total cost per HPV related precancer and cancer type. (b) Attributable fraction of total cost of HPV related precancers and cancers per cost type; direct costs and indirect costs. (c) Attributable fraction of total cost of HPV related precancers and cancers per gender. (d) Attributable fraction of total cost per HPV related precancers and cancers for females. (e) Attributable fraction of total cost per HPV related precancers and cancers for males.

The total annual cost for the HR HPV-related diseases considered in this study was estimated at €93.7 million (between €89.9 million and €97.7 million estimated with lower and upper bounds) at (Table 5) of which cervical cancer represented the majority of the economic burden at 62% (Fig 3a). Direct costs were estimated at €31.1 million, of which HR HPV-attributable inpatient care and outpatient care represented €20.4 million and €10.7 million, respectively. Indirect costs were estimated at €62.6 million, of which HR HPV-attributable morbidity and premature death accounted for €26.6 and €36 million, respectively. Indirect costs (productivity costs) represented the majority of the economic burden at 67% (Fig 3b).

**Table 5:** Total annual HR HPV-attributable costs in Sweden in 2006, presented in €1000.

Type of cost	Cervix	Vulva	Vagina	Anus		Tonsil		Base of tongue		Penis	Base case cost	Lower bound	Upper bound	Lowest range	Highest range	Total annual cost
	F	F	F	M	F	M	F	M	F	M	M,F	M,F	M,F	M,F	M,F	M,F
*Direct costs*	17 854	2 388	1 784	1 881	3 431	1 139	677	454	371	1 100	31 080	29 857	32 155	25 657	37 719	40 571
Inpatient care	10 993	1 491	730	1 521	2 627	901	591	380	320	872	20 427	19 387	21 346	16 647	25 617	27 189
Outpatient care	6 860	897	1 055	360	804	238	86	74	51	228	10 653	10 470	10 809	9 011	12 102	13 383
*Indirect costs*	40 577	2 202	2 222	2 202	4 596	5 322	1 578	1 569	837	1 528	62 632	59 488	65 554	50 230	78 483	83 646
Mortality (premature death)*	25 520	1 204	1 658	1 185	1 563	2 280	489	814	489	831	36 031	34 489	37 505	28 705	44 012	46 842
Short term sick leave	2 263	123	64	143	396	468	131	95	61	61	3 806	3 550	4 039	3 079	5 069	5 452
Long-term sick leave	7 224	392	204	456	1 266	1 495	419	303	195	196	12 149	11 485	12 749	9 794	15 476	16 495
Early retirement	5 571	482	296	418	1 371	1078	539	358	93	440	10 646	9 965	11 261	8 651	13 927	14 857
*Total annual costs*	58 431	4 590	4 007	4 083	8 027	6 461	2 255	2 023	1 209	2 628	93 712	89 345	97 709	75 887	116 202	124 218

F = Females, M = Males,

*3% discount rate

When examined by gender, females represented the majority of the economic burden, with 84% (Fig 3c). For females, total annual costs for all HR HPV-attributable cancers were estimated at €78.5 million; morbidity and mortality contributed €21 million and €30.9 million, respectively. All gynecological cancers combined represented the majority of the economic burden among females, accounting for 86% (Fig 3d) and contributing an estimated €67 million, with cervical cancer representing the majority of that burden (€58.4 million). The total direct costs for cervical cancer were €17.8 million, whereas corresponding costs for cervical dysplasia and CIS were estimated at €7.3 million (74% for outpatient care and 26% for inpatient care). Total annual costs for vulvar cancer were estimated at €4.6 million; total annual direct costs for this cancer were €2.4 million, including direct costs of €1.2 million for vulvar dysplasia and CIS (38% outpatient costs and 62% inpatient costs). The total annual costs for vaginal cancer were estimated at €4 million. Direct costs were €1.8 million, of which €1.3 million (20% outpatient costs and 80% inpatient costs) were represented by vaginal dysplasia and CIS. Indirect costs for all gynecological cancers were estimated at €45 million, of which morbidity and premature mortality was estimated at €28.4 million and €16.6 million, respectively. Cervical cancer had the largest indirect cost, at €40.6 million. Total annual costs for anal cancer among females were estimated at €8 million, with direct costs of €3.4 million; anal dysplasia and CIS accounted for €0.8 million of this amount (57% outpatient costs and 43% inpatient costs). Total annual costs for oropharyngeal cancer among females were estimated at €3.4 million (tonsillar cancer: €2.3 million and base of tongue cancer: €1.2 million).

For males, oropharyngeal cancer was represented the majority of the economic burden, accounting for 56% (Fig 3e) with total annual societal costs of €8.5 million (tonsillar cancer: €6.5 million and base of tongue cancer: €2 million). This was followed by anal cancer at an estimated €4.1 million, which included direct costs of €1.9 million, of which anal dysplasia and CIS accounted for €0.7 million (67% outpatient care and 33% inpatient care). Total annual societal costs for penile cancer were estimated at €2.6 million, with direct costs of 1.1 million, of which CIS accounted for €0.07 million (56% outpatients care and 44% inpatient care). Total annual societal costs for all HR HPV-attributable cancers in males were estimated at €15.2 million: morbidity represented €5.5 million and mortality due to premature death €5.1 million.

When altering the ranges of HR HPV prevalence to the lowest and highest ranges reported in the articles from the literature review, total annual societal cost for all HR HPV-related diseases and both genders in this study was estimated at €75.9 million and €116.2 million, respectively. The total annual societal costs for all the diseases in this study were estimated at €124.2 million.

## Discussion

This is the first Swedish study to evaluate the annual economic burden of HR HPV-related precancers and cancers in Sweden, including health care costs and lost productivity due to sick leave, early retirement, and premature mortality. We estimated the annual societal cost of these cancers in 2006 to be almost €94 million, and this cost ranged from €76 million to €116 million when using the lower and upper bounds of prevalence rates for the literature review. Health care costs (direct costs) and lost productivity (indirect costs) represented 33% and 67% of the total annual cost, respectively. The two main cost drivers were lost productivity due to premature mortality and inpatient health care costs, which represented 38% and 22% of the total annual cost, respectively. Cervical cancer was the diagnosis that contributed the largest economic burden (62%), with an estimated cost of €58.4 million. All gynecological cancers combined had an estimated cost of €67 million and represented 71% of the total annual cost.

For both genders, costs for anal and oropharyngeal cancer were estimated at €12.1 million and €11.9 million, respectively; both representing 26% of the total annual cost. Least costly was penile cancer at €2.6 million (3%). Among males, oropharyngeal cancer was the most costly disease with an estimated total of €8.5 million. Using the lowest and highest HR HPV prevalence rates from the literature review altered this cost between €6.9 million and €15.5 million. The largest cost driver in oropharyngeal cancer alone was morbidity and premature death, representing almost 80% of the total annual cost of the disease. Oropharyngeal cancer occurs primarily in white males aged 40–55 years [64] and has a high mortality rate [65]. Detection usually occurs at late stages and subsequent therapy can result in lifelong oral complications, which could explain the high number of registered sick days due to this cancer, and the subsequent high costs of lost productivity we found in this study. Indeed, costs for morbidity due to oropharyngeal cancer were estimated between €3.1 million and €6.9 million, respectively, when using the lowest and highest prevalence rates from the literature review.

Given these numbers, there is a real risk that lost productivity costs among males will increase in future, non-vaccinated generations of males. Indeed, the prevalence of HPV-positive oropharyngeal cancer has been increasing in the United States, Western Europe, and Australia [10, 11] and ranges from 45–90% depending on the study and the detection method [10]. Research has shown that the HPV-4 vaccine is highly efficacious in reducing infection with HPV6, 11, 16, and 18, and in reducing the subsequent development of diseases related to these HPV types in both females and males [13–15]. Data presented in a recent study showed that vaccine-induced HPV16- and 18-antibodies were detected in the oral mucosa of two different types of oral specimen [66]. Although there is a need for further research on long-term immune responses and other aspects of HPV vaccination, the successful prevention of HPV-related cancer depends on national, long-term protection against infection. Modifying the HPV Immunization Programme in Sweden to include young males has the potential to prevent HPV-related diseases like those of the external genital skin and anal canal [13–15] in males. Furthermore, since the HPV vaccine has proven to induce a robust humoral immune response to HPV, there is no reason these vaccines could not be co-opted for the prevention of oropharyngeal cancer. The current HPV Immunization Programme includes only young females, and coverages above 50% in such programs are suggested to give herd immunity [67]. Since the introduction of the HPV vaccine in Sweden, the incidence of anogenital warts has decreased among both the vaccinated female population and the unvaccinated male population, suggesting that herd immunity has been achieved [68, 69]. However, among males having sex with males, herd immunity has not yet been proven, which is why a recent modeling study recommended targeted prevention strategies to reach this population [70].

Although estimated costs from this cost-of-illness study are substantial, it is important to note that these costs are likely to be underestimated for several reasons. Firstly, the HR HPV prevalence rates used in our calculations were taken from clinical studies with different study populations and detection methods, which most likely impacted the results. Secondly, we only included costs related to health care visits where the HR HPV-related cancers under study were registered as main diagnosis; if an HR HPV-related cancer was registered as a secondary diagnosis the case was excluded. Thirdly, we did not include costs resulting from primary care, since there is no national register that includes this information in Sweden. Therefore, individuals seeking health care for symptoms related to the cancers under study or for follow-up procedures in a primary care setting are not included. Also, the lack of a national register for other costs, such as palliative care occurring outside a hospital setting, or patient transportation to different municipalities, means that these costs were also not taken into account. Due to a lack of data, we also have no costs related to patients who received informal care from family members, which is expected to impact cost estimates. Nor did we include the costs of screening for cervical cancer or HPV vaccination of young females, which are both population-based programs that are likely to increase the total annual costs significantly. In our previous study, the costs for cervical cancer screening alone were estimated at approximately €68 million in 2009 [18]; this would more than double the total annual cost for cervical cancer we report in the present study.

In 2008, the costs of HPV Immunization Programme were estimated at €26 million, with a cost per dose of around €100 [71]. At present, the official direct medical costs for the vaccine are approximately €15 per dose [72]. The HPV vaccine, when given in a two-dose schedule within 1 year, would lead to annual costs of approximately €3 million based on a population of 100 000 young females and males of vaccinating age (10–12 years). However, the costs of distribution and administration of vaccines by school nurses should also be added to this. Nevertheless, the direct medical costs for the vaccination of both boys and girls are minor in relation to the overall economic burden for the HR HPV-related diseases in this study.

When comparing the cost of the HR HPV-related cancers included in our study with those reported in other cost-of-illness studies performed in Sweden, results show that HPV infection can cause clinical conditions with high lost productivity costs. This could be explained by morbidity and premature mortality affecting individuals of working age (below age 65). Lost productivity is often excluded from economic analyses, which could lead to an underestimation of the societal benefits of interventions and/or treatment. However, there are several challenges in reliably when calculating and estimating lost productivity. In this study, we used the human capital method to estimate the value of lost productivity. This method is grounded in economic theory and is based on the assumption that companies either employ labor until the marginal value of an employee’s work productivity is equal to the marginal cost of labor or the employee’s gross wage [73]. A common criticism of this method is that it discriminates against individuals above retirement age and overestimates the cost of lost production because it disregards potential work replacement, which eventually results in diminished production losses (i.e., the friction cost method) [74]. The criticism is especially relevant for cervical cancer, since 70% of the women who died from this disease in 2006 where above 65 years of age. On the other hand, the majority of individuals with oropharyngeal cancer were of working age, making for a high lost productivity cost.

Our proportion of the total annual cost made up by indirect costs was equivalent to those reported in other cost-of-illness studies on breast cancer [75], depression [76], and brain tumors [77] in Sweden. When comparing only direct costs without considering the costs of the screening program, the costs of our HR HPV-related cancers were equivalent to those of multiple sclerosis [78] and brain tumors in Sweden [77]. Cost-of-illness studies attempt to estimate the economic burden a specific disease places upon society. Such studies should not be considered economic evaluations, since they do not investigate outcomes. Since cost-of-illness studies do not assess the health gains from any specific intervention, they cannot give guidance on how resources should be allocated to achieve maximum health gains. Instead, cost-of-illness studies should act as point of reference for further economic evaluations. Prevalence-based cost-of-illness studies, such as this one presenting the amount of money Sweden spends annually caring for patients with HPV-related diseases, are useful to health policy makers for planning and budget decisions. This study also demonstrates how costs are distributed between direct and indirect costs within the diseases and where the major costs occur, which gives policy makers new insight into where resources are spent. However, economic evaluations (e.g., cost-effectiveness analysis, cost-utility analysis, and cost-benefit analysis) are the correct methodologically to use when trying to prioritize public investments into different interventions, treatments, or investments, because they account for the changes in survival and quality of life that are associated with these interventions. Interventions like screening and HPV vaccination are aimed at preventing the acquisition of HPV infection and subsequent disease. To-date, there are several well-known cost-effectiveness analyses assessing HPV vaccination programs and cervical cancer screening [79–81]. However, there has been no cost-effectiveness analysis published so far that combines vaccination and screening and considers all HPV-related diseases with associated costs and outcomes for both genders.

### Conclusions

In conclusion, HR HPV-related cancers constitute a major public health issue. With current evidence on HPV vaccines against HPV16 and18, which are the most common types associated with malignant transformation of the cervix and oropharynx, the rapid increase in the incidence of HPV-positive oropharyngeal cancer among young males should be addressed by including boys into the HPV Immunization Programme in Sweden. A retrospective analysis of current data for the incidence of oropharyngeal cancer would be beneficial, although time-consuming. Regardless, the value of introducing HPV vaccination in boys in the near future should be considered, given the predictions that oropharyngeal cancer will surpass cervical cancer as the most common HPV-related cancer in the next decades. The results from this study could act as a point of reference for future economic evaluations on the overall benefits of HPV vaccination in females and males in Sweden and are useful to health policy makers when deciding whether to include males into the HPV Immunization Programme.

## Supporting information

S1 Table Diagnoses included in our cost estimates. (DOCX)

S1 Fig Information flow through the different phases in the systematic literature review. (TIF)
