# Correction: The economic burden of human papillomavirus-related precancers and cancers in Sweden

**DOI:** 10.1371/journal.pone.0200554

**Published:** 2018-07-09

**Authors:** Ellinor Östensson, Maria Silfverschiöld, Lennart Greiff, Christine Asciutto, Johan Wennerberg, Marie-Louise Lydrup, Ulf Håkansson, Pär Sparén, Christer Borgfeldt

The sixth author’s name is spelled incorrectly. The correct name is: Marie-Louise Lydrup. The correct citation is: Östensson E, Silfverschiöld M, Greiff L, Asciutto C, Wennerberg J, Lydrup M-L, et al. (2017) The economic burden of human papillomavirus-related precancers and cancers in Sweden. PLoS ONE 12(6): e0179520. https://doi.org/10.1371/journal.pone.0179520.

There are errors in the author affiliations. The affiliations should appear as shown here:

Ellinor Östensson^1,2^, Maria Silfverschiöld^3^, Lennart Greiff^3^, Christine Asciutto^4^, Johan Wennerberg^3^, Marie-Louise Lydrup^5^, Ulf Håkansson^6^, Pär Sparén^1^, Christer Borgfeldt^4^

1 Department of Medical Epidemiology and Biostatistics, Karolinska Institutet, Stockholm, Sweden, 2 Department of Women´s and Children´s Health, Division of Obstetrics and Gynecology, Karolinska Institutet, Stockholm, Sweden, 3 Department of Otorhinolaryngology Head & Neck Surgery, Skåne University Hospital, Lund University, Lund, Sweden, 4 Department of Obstetrics and Gynecology, Skåne University Hospital, Lund University, Lund, Sweden, 5 Department of Surgery, Skåne University Hospital, Lund University, Malmö, Sweden, 6 Department of Urology, Skåne University Hospital, Lund University, Malmö, Sweden.

## References

[1] Östensson E, Silfverschiöld M, Greiff L, Asciutto C, Wennerberg J, Lydryp M-L, et al (2017) The economic burden of human papillomavirus-related precancers and cancers in Sweden. PLoS ONE 12(6): e0179520 https://doi.org/10.1371/journal.pone.0179520 28651012 10.1371/journal.pone.0179520 PMC5484479

