# Supplementary material for: The economic burden of human papillomavirus-related precancers and cancers in Sweden
Source: PLoS One. 2017 Jun 26;12(6):e0179520. doi: 10.1371/journal.pone.0179520 (PMC5484479; doi:10.1371/journal.pone.0179520)
Supplement: S1 Table — (DOCX) [file pone.0179520.s001.docx]

**S1 Table. Diagnoses included in our cost estimates.**

| **Site** | **ICD-code** | **Diagnosis** |
| --- | --- | --- |
| Cervix | N87 | Cervical dysplasia |
|  | D06 | Cervical carcinoma *in situ* |
|  | C53 | Cervical cancer |
| Vulva | N90 | Vulvar dysplasia |
|  | D07.1 | Vulvar carcinoma *in situ* |
|  | C51 | Vulvar cancer |
| Vagina | N89 | Vaginal dysplasia |
|  | D07.2 | Vaginal carcinoma *in situ* |
|  | C52 | Vaginal cancer |
| Anus | K62 | Anal dysplasia |
|  | D01.3 | Anal carcinoma *in situ* |
|  | C21 | Anal cancer |
| Penis | D07.4 | Penile carcinoma *in situ* |
|  | C60 | Penile cancer |
| Oropharynx | C09 | Tonsillar cancer |
|  | C01 | Base of tongue cancer |
