# Supplementary figures and images for: The economic burden of human papillomavirus-related precancers and cancers in Sweden
Source: PLoS One. 2017 Jun 26;12(6):e0179520. doi: 10.1371/journal.pone.0179520 (PMC5484479; doi:10.1371/journal.pone.0179520)

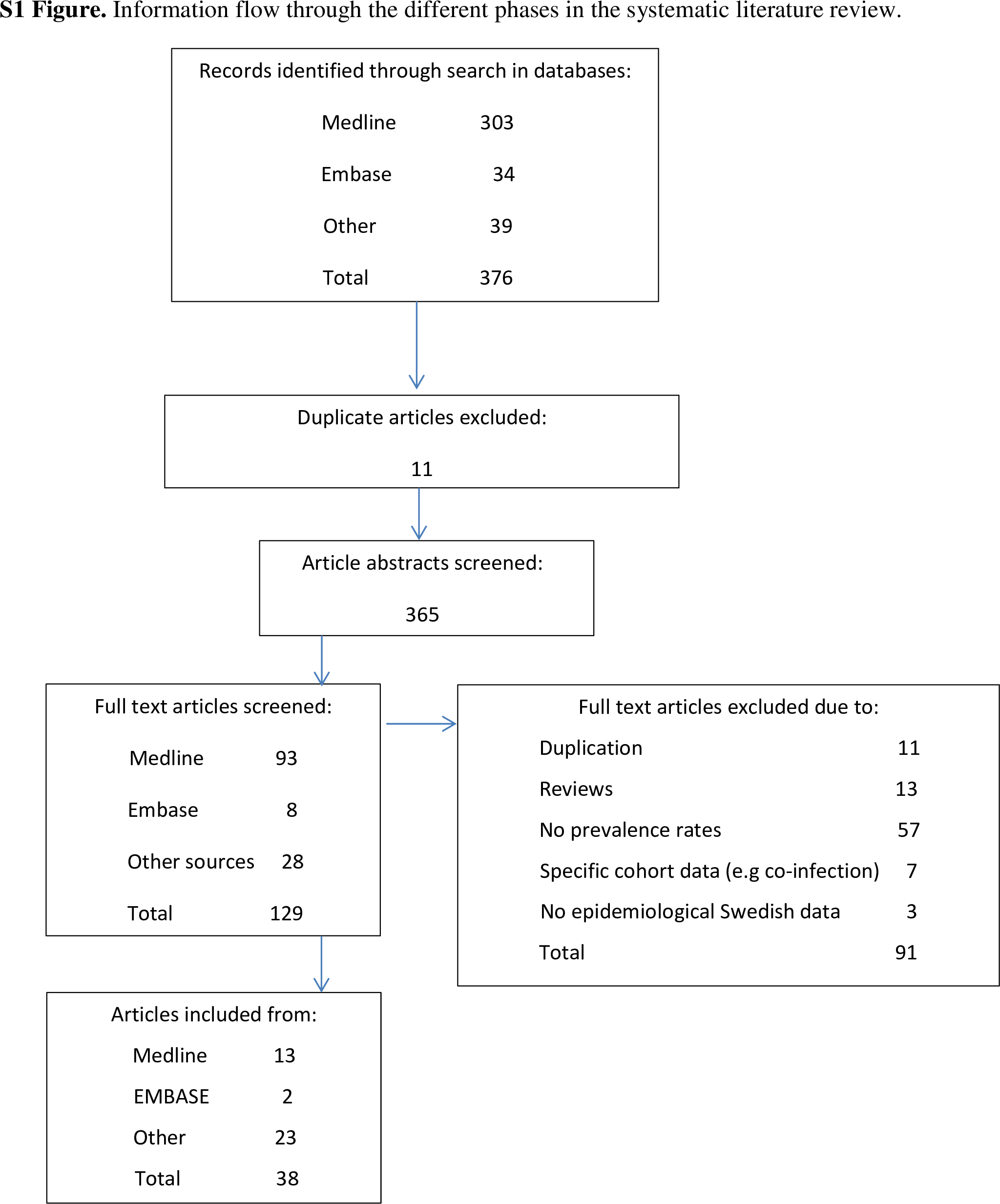

Supplement: S1 Fig — (TIF) [file pone.0179520.s002.tif]
